# Sex-based disparities and in-hospital outcomes of patients hospitalized with atrial fibrillation with and without dementia

**DOI:** 10.1016/j.ahjo.2023.100266

**Published:** 2023-02-03

**Authors:** Nischit Baral, Joshua D. Mitchell, Neelum T. Aggarwal, Timir K. Paul, Amith Seri, Abdul K. Arida, Parul Sud, Arvind Kunadi, Krishna P. Bashyal, Nisha Baral, Govinda Adhikari, Melissa Tracy, Annabelle Santos Volgman

**Affiliations:** a Department of Internal Medicine, McLaren Flint/Michigan State University College of Human Medicine, Flint, MI, USA; b Department of Internal Medicine, Division of Cardiology, Washington University in St. Louis, Saint Louis, MO, USA; c Department of Neurological Sciences, Rush Alzheimer's Disease Center, Rush University-Rush Medical College, Chicago, IL, USA; d Department of Cardiovascular Sciences, University of Tennessee College of Medicine, Nashville, TN, USA; e Department of Microbiology, Manipal College of Medical Sciences, Pokhara, Nepal; f Department of Medicine, University of Arizona College of Medicine, Tucson, AZ, USA; g Department of Internal Medicine, Division of Cardiology, RUSH University-Rush Medical College, Chicago, IL, USA

**Keywords:** Atrial fibrillation, Dementia, In-hospital mortality, Cohort study, National Inpatient Sample

## Abstract

**Study objective:**

We sought to evaluate the sex-based disparities and comparative in-hospital outcomes of principal AF hospitalizations in patients with and without dementia, which have not been well-studied.

**Design:**

This is a non-interventional retrospective cohort study.

**Setting and participants:**

We identified principal hospitalizations of AF in the National Inpatient Sample in adults (≥18 years old) between January 2016 and December 2019.

**Main outcome measure:**

In-hospital mortality.

**Results:**

Of 378,230 hospitalized patients with AF, 49.2 % (n = 186,039) were females and 6.1 % (n = 22,904) had dementia. The mean age (SD) was 71 (13) years. Patients with dementia had higher odds of in-hospital mortality {adjusted odds ratio (aOR): 1.48, 95 % confidence interval (CI): 1.34, 1.64, p < 0.001} and nontraumatic intracerebral hemorrhage (aOR: 1.60, 95 % CI: 1.04, 2.47, p = 0.032), but they had lower odds of catheter ablation (0.39, 95 % CI: 0.35, 0.43, p < 0.001) and electrical cardioversion (aOR: 0.33, 95 % CI: 0.31, 0.35, p < 0.001). In patients with AF and dementia, compared to males, females had similar in-hospital mortality (aOR: 1.00, 95 % CI: 0.93, 1.07, p = 0.960), fewer gastrointestinal bleeds (aOR: 0.92, 95 % CI: 0.85, 0.99, p = 0.033), lower odds of getting catheter ablation (aOR: 0.79, 95 % CI: 0.76, 0.81, p < 0.001), and less likelihood of getting electrical cardioversion (aOR: 0.78, 95 % CI: 0.76, 0.79, p < 0.001).

**Conclusions:**

Patients with AF and dementia have higher mortality and a lower likelihood of getting catheter ablation and electrical cardioversion.

## 1 Introduction

Atrial fibrillation (AF) and dementia share a complex relationship beyond comorbidity. AF is independently associated with an increased risk of dementia, even after adjusting for stroke [1], [2], [3]. The relationship between AF and dementia is explained by changes in brain perfusion due to an abnormal rhythm, bleeding due to anticoagulation, or embolic events [1]. In-hospital outcomes of the population hospitalized with AF and dementia have not been well studied in the literature [1], [2], [3], [4]. The sex-based difference in the outcomes of principal AF hospitalizations with and without dementia is also not well known [1], [2], [3], [4]. The Atherosclerosis Risk in Communities (ARIC) study and Atherosclerosis Risk in Communities-Neurocognitive Study (ARIC-NCS) demonstrated an increased risk of incident dementia in patients with AF and an increased likelihood of comorbid dementia in incident AF [2], [3], [4], [5]. Both studies have investigated dementia in community-based settings; however, studies highlighting differences in outcomes of AF based on sex and comorbidity of dementia in hospital settings are lacking [2], [3], [4], [5]. To close this gap, we aimed to study sex-based and dementia-based differences in the in-hospital outcomes of principal AF hospitalizations using data from the National Inpatient Sample (NIS).

## 2 Material and methods

Our study was waived from ethical approval and informed consent by our local institutional review board because NIS is publicly available de-identified data. We followed the Strengthening the Reporting of Observational Studies in Epidemiology (STROBE) guideline in reporting our study.

### 2.1 Study design

We retrospectively identified all principal hospitalizations of AF in adults (≥18 years old) in the 2016–2019 NIS, Healthcare Cost and Utilization Project (HCUP), and Agency for Healthcare Research and Quality [6]. The NIS is the largest administrative database in the US. Information about the NIS's design, recruitment, and logistics can be found on the HCUP website (https://www.hcup-us.ahrq.gov) [6]. Within the NIS, the unweighted sample size refers to a smaller sample from approximately 20 % of all US hospitalizations. It contains clinical data on inpatient diagnoses (both primary and secondary diagnoses) and procedures from approximately 7 million hospitalizations annually. Principal diagnoses or procedures are the main diagnosis or procedures for which the patient was primarily admitted to the hospital. The weighted sample is more extensive and provides a national estimate. We used only the unweighted sample for our analysis. More details regarding the NIS can be found on the HCUP website [6].

### 2.2 Study population, variables, and outcomes

Patients with a principal diagnosis of AF (diagnosis variable DX1) and dementia as a secondary diagnosis (diagnosis variable DX2 to DX25) were identified using the International Classification of Diseases, Tenth Revision, and Clinical Modification (ICD-10-CM). The ICD-10-CM diagnosis and procedural codes are shown in eTable 1 in the Supplement.

We extracted sociodemographic data, including age, gender, race, primary payer, household income, hospital location, teaching-hospital status, and comorbidities associated with the principal diagnosis as per the HCUP website [6]. We used the Charlson comorbidity index (CCI) to assess and account for comorbidities that could influence survival or other outcomes (stratified 0, 1, 2, ≥3).

The primary outcome of interest was in-hospital mortality, which was calculated as the total number of deaths (NIS variable “DIED”) in principal AF hospitalizations (numerator) divided by the total number of hospitalizations for the same (denominator). The secondary outcomes of interest were the occurrence of gastrointestinal (GI) bleeding, nontraumatic intracerebral hemorrhages (including subarachnoid hemorrhage {SAH}), the in-hospital procedure of intubation and mechanical ventilation, catheter ablation, and electrical cardioversion. The outcomes of mechanical ventilation, catheter ablation, and electrical cardioversion were captured using the ICD-10 principal or secondary procedural code from NIS, as shown in eTable 1 in Supplement.

### 2.3 Statistical analysis

We evaluated the data for outliers and tested the distribution of the outcomes. Characteristics of the unweighted study sample included means, medians, interquartile ranges, frequencies, and percentages. We used Chi^2^ or Fisher's exact test for categorical variables (e.g., mortality, race, and comorbidity index categories) to examine possible group differences (based on sex and dementia). We used a student *t*-test to compare group differences among continuous variables (age and length of stay).

Univariable and multivariable logistic regression analyses examined whether sex and dementia were associated with changes in in-hospital mortality, GI bleeding, intracerebral hemorrhage, and the requirement for mechanical ventilation, catheter ablation, and electrical cardioversion. Univariable logistic regression further explored the relationship between the outcomes of interest and sociodemographic characteristics (e.g., age, sex, race, hospital region, and national quartiles for household income) and the Charlson comorbidity index. The variables included in the multivariable regression model were dementia, sex, and covariates with a p-value of 0.10 or lower in the unadjusted regressions using forward selection regression methods of entry. Two-sided p-value <0.05 was considered statistically significant. Data were analyzed in August 2022. We first used STATA's “mistable summarize” command to perform multiple imputations to calculate the missing observations. Missing observations were reported, and multiple imputations were performed if the missing observations were >5 %. Since the total missing observations were 4 % of the final observations, we did not perform multiple imputations. We reported the bivariate analysis in the unweighted sample. In this analysis, the precision of the study's estimate is indicated by a 95 % confidence interval (95 % CI), and p-values are employed to interpret the results. All analyses were performed in STATA 17.0 (Stata-Corp LP, College Station, Texas).

## 3 Results

### 3.1 Baseline characteristics

**Fig. 1:**
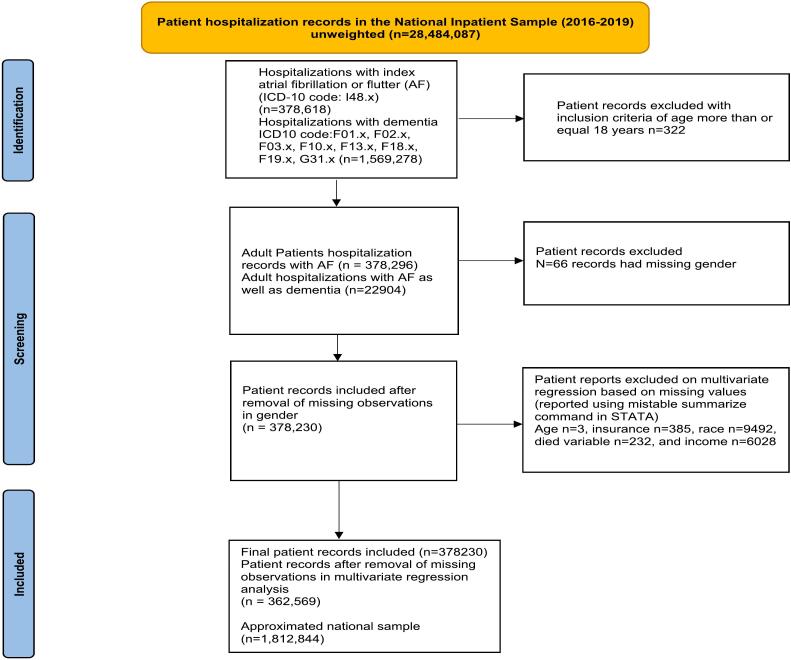
Patient identification flowchart. Abbreviations: ICD: International Classification of Disease, AF: atrial fibrillation/flutter.

There were 378,230 principal hospitalizations with AF during the study period from 2016 to 2019. A flow diagram of the included studies and missing observations is shown in Fig. 1. Among index AF admissions, 49.2 % (n = 186,039) were females, 81.7 % were White, and 32 % had a Charlson comorbidity index (CCI) of 3 or more. The mean age was 71 ± 13 years, with females older than men (mean age: 74 vs. 67; p < 0.001). Among principal AF admissions, 6.1 % (n = 22,904) had dementia, 0.85 % died in hospital, 0.81 % had a GI bleed, 0.05 % had a nontraumatic intraparenchymal hemorrhage, 19.7 % received electrical cardioversion, and 5.5 % received catheter ablation. Table 1, Table 2.

**Table 1:** Baseline characteristics of the unweighted sample for adults with AF comparing males and females from NIS 2016–2019 in US inpatient admissions.

Variable	Total N = 378,230	Male n = 192,191	Female n = 186,039	p-Value
Age (years)	71 (±13)	67 (±13)	74 (±12)	<0.001
Race: White	301,156 (81.7 %)	153,001 (81.7 %)	148,115 (81.7 %)	<0.001
Black	31,131 (8.4 %)	15,842 (8.5 %)	15,289 (8.4 %)	
Hispanic	21,899 (6 %)	11,153 (6 %)	10,746 (6 %)	
Asian	5619 (1.5 %)	2667 (1.4 %)	2952 (1.6 %)	
Medicare	259,561 (68.7 %)	114,050 (59.4 %)	145,511 (78.2 %)	<0.001
Medicaid	23,474 (6.2 %)	14,613 (7.6 %)	8861 (4.8 %)	
Private/HMO insurance	77,318 (20.5 %)	50,504 (26.3 %)	26,814 (14.4 %)	
Self-paying	9134 (2.4 %)	6366 (3.3 %)	2768 (1.5 %)	
Region: Northeast	74,767 (20 %)	38,352 (20 %)	36,415 (19.6 %)	<0.001
Midwest	91,850 (24.2 %)	46,260 (24 %)	45,590 (24.5 %)	
South	153,863 (40.7 %)	77,831 (40.5 %)	76,032 (40.9 %)	
West	57,750 (15.2 %)	29,748 (15.5 %)	28,002 (15 %)	
Charlson comorbidity index				
0	86,007 (22.7 %)	44,068 (22.9 %)	41,939 (22.5 %)	<0.001
1	97,314 (25.7 %)	47,928 (25 %)	49,386 (26.6 %)	
2	74,059 (19.6 %)	36,881 (19.1 %)	37,178 (20 %)	
3 or higher	120,850 (32 %)	63,314 (32.9 %)	57,536 (30.9 %)	
Annual income				<0.001
1–45,999	101,716 (27.3 %)	0,662 (26.9 %)	51,054 (27.8 %)	
46,000–58,999	100,847 (27 %)	50,761 (27 %)	50,086 (27.2 %)	
59,000–78,999	92,746 (25 %)	47,269 (25 %)	45,477 (24.8 %)	
79,000 or more	76,898 (20.7 %)	40,014 (21.2 %)	36,884 (20.1 %)	
AMI	43,015 (11.4 %)	24,890 (13 %)	18,125 (9.7 %)	<0.001
CHF	164,325 (43.5 %)	84,741 (44.1 %)	79,584 (42.8 %)	<0.001
PVD	38,120 (10.1 %)	22,322 (11.6 %)	15,978 (8.5 %)	<0.001
CEVD	16,954 (4.5 %)	7864 (4.1 %)	9090 (4.9 %)	<0.001
COPD	95,638 (25.3 %)	45,865 (23.9 %)	49,773 (26.8 %)	<0.001
Rheumatoid disease	11,657 (3.1 %)	3289 (1.7 %)	8368 (4.5 %)	<0.001
Hemiplegia	1532 (0.4 %)	805 (0.4 %)	727 (0.4 %)	0.175
Kidney disease	71,933 (19 %)	37,528 (19.5 %)	34,405 (18.5 %)	<0.001
Cancer	14,049 (3.7 %)	7881 (4.1 %)	6168 (3.3 %)	<0.001
Peptic ulcer	2624 (0.7 %)	1298 (0.7 %)	1326 (0.7 %)	0.170
Mild liver disease	9669 (2.6 %)	6204 (3.2 %)	3465 (1.9 %)	<0.001
Diabetes mellitus	71,166 (18.8 %)	37,247 (19.4 %)	33,919 (18.2 %)	<0.001
Diabetes with complications	38,005 (10.1 %)	20,312 (10.6 %)	17,693 (9.5 %)	<0.001
Moderate/severe liver disease	1484 (0.4 %)	973 (0.5 %)	511 (0.3 %)	<0.001
Metastatic cancer	5739 (1.5 %)	3363 (1.8 %)	2377 (1.3 %)	<0.001
AIDS	351 (0.1 %)	278 (0.1 %)	73 (0.04 %)	<0.001
In-hospital mortality	3197 (0.9 %)	1458 (0.8 %)	1739 (0.9 %)	<0.001
Length of stay (days)	3.4 (± 3.7)	3.2 (± 3.7)	3.5 (± 3.6)	<0.001
Catheter ablation	20,949 (5.5 %)	12,417 (6.5 %)	8532 (4.6 %)	<0.001
Electrical cardioversion	74,441 (19.7 %)	43,500 (22.6 %)	30,941 (16.6 %)	<0.001
Dementia	22,904 (6.1 %)	7834 (4.1 %)	15,070 (8.1 %)	<0.001
Mechanical ventilation	2049 (0.5 %)	1189 (0.6 %)	860 (0.5 %)	<0.001
Mechanical circulatory support	65 (0.2 %)	49 (0.3 %)	16 (0.1 %)	<0.001
Cardiogenic shock	2288 (0.6 %)	1436 (0.8 %)	852 (0.5 %)	<0.001

**Table 2:** Baseline characteristics of the unweighted sample for adults with AF comparing with and without dementia NIS 2016–2019 in US inpatient admissions.

Variable	Total N = 378,230	Dementia n = 22,904	No dementia n = 355,326	p-Value
Age (years)	71 (±13)	83 (±7)	70 (±13)	<0.001
Sex: Female	186,039	15,070 (65.8 %)	170,969 (48.1 %)	<0.001
Male	192,191	7834 (34.2 %)	184,357 (51.9 %)	<0.001
Race: White	301,206 (81.7 %)	18,123 (81 %)	283,083 (81.7 %)	<0.001
Black	31,136 (8.4 %)	1889 (8.4 %)	29,247 (8.4 %)	
Hispanic	21,903 (5.9 %)	1461 (6.5 %)	20,442 (5.9 %)	
Asian	5619 (1.5 %)	396 (1.8 %)	5223 (1.5 %)	
Medicare	259,607 (68.7 %)	21,235 (92.8 %)	238,372 (67.1 %)	<0.001
Medicaid	23,477 (6.2 %)	349 (1.5 %)	23,128 (6.5 %)	
Private/HMO	77,330 (20.5 %)	977 (4.3 %)	76,353 (21.5 %)	
Self-paying	9138 (2.4 %)	89 (0.4 %)	9049 (2.6 %)	
Region: Northeast	74,776 (19.8 %)	4341 (19 %)	70,435 (19.8 %)	<0.001
Midwest	91,860 (24.3 %)	5129 (22.4 %)	86,731 (24.4 %)	
South	153,895 (40.7 %)	9836 (42.9 %)	144,059 (40.5 %)	
West	57,765 (15.3 %)	3600 (15.7 %)	54,165 (15.2 %)	
Charlson comorbidity index				<0.001
1	97,328 (25.7 %)	4125 (18 %)	93,203 (26.2 %)	
2	74,071 (19.6 %)	5784 (25.3 %)	68,287 (19.2 %)	
3	120,871 (32 %)	12,997 (56.7 %)	107,874 (30.4 %)	
Annual income				
1–45,999	101,735 (27.3 %)	6383 (28.2 %)	95,352 (27.3 %)	0.004
46,000–58,999	100,866 (27.1 %)	5975 (26.4 %)	94,891 (27.1 %)	
59,000–78,999	92,759 (24.9 %)	5536 (24.5 %)	87,223 (24.9 %)	
79,000 or more	76,908 (20.7 %)	4709 (20.8 %)	72,199 (20.7 %)	
AMI	43,022 (11.4 %)	2880 (12.6 %)	40,142 (11.3 %)	<0.001
CHF				
PVD	38,127 (10.1 %)	2391 (10.4 %)	35,736 (10.1 %)	0.063
CEVD	16,958 (4.5 %)	2139 (9.3 %)	14,819 (4.2 %)	<0.001
COPD	95,655 (25.3 %)	5483 (24 %)	90,172 (25.4 %)	<0.001
Rheumatoid disease	11,662 (3.1 %)	668 (2.9 %)	10,994 (3.1 %)	0.134
Hemiplegia	1533 (0.4 %)	168 (0.7 %)	1365 (0.4 %)	<0.001
Kidney disease	71,945 (19 %)	6076 (26.5 %)	65,869 (18.5 %)	<0.001
Cancer	14,056 (3.7 %)	689 (3 %)	13,367 (3.8 %)	<0.001
Peptic ulcer	2625 (0.7 %)	179 (0.8 %)	2446 (0.7 %)	0.100
Mild liver disease	9669 (2.6 %)	316 (1.4 %)	9353 (2.6 %)	<0.01
Diabetes mellitus	71,173 (18.8 %)	3973 (17.3 %)	67,200 (18.9 %)	<0.001
Diabetes with complications	38,008 (10.1 %)	2584 (11.3 %)	35,424 (10 %)	<0.001
Moderate/severe liver disease	1484 (0.4 %)	56 (0.2 %)	1428 (0.4 %)	<0.001
Metastatic cancer	5741 (1.5 %)	232 (1 %)	5509 (1.6 %)	<0.001
In-hospital mortality	3197 (0.9 %)	550 (2.4 %)	2647 (0.8 %)	<0.001
Length of stay (days)	3.4 (± 3.7)	4.8 (± 6.4)	3.3 (± 3.4)	<0.001
Catheter ablation	20,949 (5.5 %)	432 (1.9 %)	20,517 (5.8 %)	<0.001
Electrical cardioversion	74,452 (19.7 %)	1451 (6.3 %)	73,001 (20.5 %)	<0.001
Mechanical ventilation	2049 (0.5 %)	137 (0.6 %)	1912 (0.5 %)	0.227
Cardiogenic shock	2288 (0.6 %)	136 (0.6 %)	2152 (0.6 %)	0.860
Gastrointestinal bleed	3046 (0.8 %)	290 (1.3 %)	2756 (0.8 %)	<0.001
Intracerebral hemorrhage	194 (0.1 %)	32 (0.1 %)	162 (0.1 %)	<0.001

### 3.2 Bivariate analysis based on sex and dementia

Among index AF, compared to males, females had a higher rate of dementia (8.1 % vs. 4.1 %, p < 0.001), higher in-hospital mortality (0.9 % vs. 0.8 %, p < 0.001), fewer catheter ablations (4.6 % vs 6.5 %, p < 0.001), and fewer cardioversions (16.6 % vs. 22.6 %, p < 0.001). Compared to AF without dementia, those with dementia were older (mean age: 83 vs. 69.7 years, p < 0.001), had higher in-hospital mortality (2.4 % vs. 0.8 %, p < 0.001), a longer length of stay (4.8 vs. 3.3 days, p < 0.001), higher comorbidities with CCI of 3 or more (56.7 % vs. 30.4 %, p < 0.001), a higher rate of GI bleeding (1.3 % vs. 0.8 %, p < 0.001), a higher rate of nontraumatic intracerebral hemorrhage (0.14 % vs. 0.05 %, p < 0.001), fewer catheter ablations (1.9 % vs 5.8 %, p < 0.001), and fewer cardioversions (6.3 % vs. 20.5 %, p < 0.001). Table 1, Table 2.

### 3.3 Multivariable regression

The final logistic regression model incorporated age, race, hospital region, income, comorbidity, insurance, and sex as the primary determinant variables. AF with dementia had higher odds of in-hospital mortality (aOR: 1.48, 95 % CI: 1.34, 1.64, p < 0.001), and nontraumatic intracerebral hemorrhage (aOR: 1.60, 95 % CI: 1.04, 2.47, p = 0.032), but lower odds of catheter ablation (0.39, 95 % CI: 0.35, 0.43, p < 0.001), and electrical cardioversion (aOR: 0.33, 95 % CI: 0.31, 0.35, p < 0.001), compared to AF without dementia (eFigs. 1, 2, and 3 in Supplement). There was no difference in gastrointestinal bleeding between patients with and without dementia.

Compared to males, females had similar in-hospital mortality (adjusted odds ratio (aOR): 1.00, 95 % CI: 0.93, 1.07, p = 0.960), fewer GI bleeds (aOR: 0.92, 95 % CI: 0.85, 0.99, p = 0.033), lower odds of getting catheter ablation (aOR: 0.79, 95 % CI: 0.76, 0.81, p < 0.001), and less likely to get electrical cardioversion (aOR: 0.78, 95 % CI: 0.76, 0.79, p < 0.001) (eFigs. 1, 2, and 3 in Supplement). We have reported the other primary and secondary outcomes based on dementia and sex in Table 3, Table 4.

**Table 3:** Unadjusted and adjusted odds ratios for in-hospital mortality among adult patients with principal AF based on sex and dementia from the NIS 2016–2019.

In-hospital mortality	Unadjusted OR	LL–UL 95 % CI	p-Value	Adjusted OR	LL–UL 95 % CI	p-Value
Age	1.05	1.05–1.06	<0.001	1.05	1.04–1.05	<0.001
Dementia (compared to without dementia)	3.28	2.98–3.60	<0.001	1.48	1.34–1.64	<0.001
Sex (ref. male)						
Female	1.23	1.15–1.32	<0.001	1.00	0.93–1.07	0.960
Race (ref. Whites)						
Blacks	1.17	1.04–1.32	0.008	1.16	1.03–1.32	0.017
Hispanics	1.00	0.86–1.17	0.947	0.97	0.83–1.14	0.714
Asians	1.21	0.92–1.60	0.172	1.07	0.80–1.43	0.639
Regional (ref. Northeast)						
Midwest	0.92	0.82–1.03	0.143	0.84	0.75–0.95	0.004
South	1.09	0.98–1.20	0.100	1.02	0.92–1.13	0.730
West	1.23	1.08–1.39	0.001	1.15	1.01–1.30	0.031
Household income by quartile (USD) (ref. less than $46,000)						
46,000–58,999	0.94	0.86–1.03	0.160	0.97	0.88–1.06	0.473
59,000–78,999	0.85	0.77–0.94	0.001	0.88	0.80–0.97	0.014
79,000 or higher	0.74	0.66–0.82	<0.001	0.76	0.68–0.85	<0.001
Charlson comorbidity category (compared to cat 0)						
1	2.54	2.11–3.05	<0.001	2.16	1.78–2.62	<0.001
2	4.21	3.51–5.04	<0.001	3.29	2.72–4.00	<0.001
3	9.80	8.29–11.59	<0.001	7.28	6.11–8.68	<0.001

Abbreviations: AF = atrial fibrillation, DM = diabetes mellitus, LL = lower limit, UP = upper limit, MCI = mild cognitive impairment, NA = Not application in multivariable regression, OR = odds ratio.

**Table 4:** Adjusted odds ratios for in-hospital outcomes among adult patients with principal AF based on sex and dementia from the NIS 2016–2019.

In-hospital outcomes	Adjusted OR	LL–UL 95 % CI	p value
Electrical cardioversion in dementia	0.33	0.31–0.35	<0.001
Electrical cardioversion in females	0.78	0.76–0.79	<0.001
Catheter ablation in dementia	0.39	0.35–0.43	<0.001
Catheter ablation in females	0.79	0.76–0.81	<0.001
Mechanical ventilation in dementia	0.94	0.78–1.13	0.502
Mechanical ventilation in females	0.86	0.79–0.95	0.002
Intracerebral hemorrhage in dementia	1.60	1.04–2.47	0.032
Intracerebral hemorrhage in females	0.98	0.73–1.33	0.913
Gastrointestinal bleed in dementia	1.06	0.93–1.21	0.360
Gastrointestinal bleed in females	0.92	0.85–0.99	0.033

## 4 Discussion

Our study highlights mainly on the outcomes associated with AF with dementia compared to AF without dementia and the sex-based disparities in the treatment or procedures received in this population. A European registry study, similar to ours, showed that females with AF are less likely to receive electrical cardioversion or catheter ablation than males [7]. Our study highlights the importance to address the higher mortality in AF hospitalization with dementia. Our study is unique in highlighting the outcomes of AF and dementia in hospitalized setting unlike previous studies in community settings [2], [4]. A 2018 review article by Pastori et al. has highlighted that patients with AF and dementia are frequently undertreated with anticoagulation, thus increasing their risk of ischemic stroke and mortality, however in our study due to limitation of NIS we couldn't comment on the use of anticoagulation in our study [8].

Our study is based on retrospective analysis of NIS in patients primarily admitted (index case) for AF and we divided this population into two cohorts, those with comorbidity of dementia and those without dementia so we couldn't directly examine the risk of dementia in AF patients. Our study didn't have a control arm of patient with and without AF to compare into the risk of dementia in these different population. Future studies are needed to understand the pathophysiology behind AF and dementia and the role of anticoagulation in the risk of dementia [3], [9], [10].

A review article by Volgman et al. highlighted that females with AF have a higher risk of stroke and mortality than males [11]. The post hoc analysis of risk factors and cardiovascular events from the Rate Control Efficacy in Permanent Atrial Fibrillation (RACE II) trial showed that cardiovascular events were not significantly higher in women after adjusting for risk factors [12]. One reason for higher mortality could be from higher risk factors in women as shown in the RACE II trial analysis [12]. Our study differs from other studies because we investigated the in-hospital mortality of dementia in principal AF admissions in US hospitals [2], [4]. Future studies are needed on the role of early rhythm control with oral antiarrhythmic medications, catheter ablation, or cardioversion in preventing dementia.

Our study has limitations, largely due to the retrospective observational nature of this study using NIS database. NIS database does not account for medications, disease follow up, disease severity, and multiple admissions. In our study, we were not able to comment on the degree of dementia which was present as a comorbidity. The degree of dementia may have impacted treatment decisions, especially if the patient is not competent to make such decisions independently. Furthermore, we were not able to comment on symptoms upon presentation and their severity along with coexistent heart failure symptoms. We could not report on them due to the limitations of NIS [6]. Due to the use of ICD-10-CM and procedures codes, we could not define the type of AF (chronic, persistent, permanent, or paroxysmal) and the treatment patients received, including anticoagulants. There is a lack of patient-level data on the duration and severity of outcomes. There is also a risk of misclassification bias and missing AF admissions in NIS database. With the use of ICD-10-CM codes, there may have been underreporting of dementia. Due to the study's observational nature, many unknown confounders could not be adjusted. Despite these limitations, our study has a large sample size and is generalizable to the US population due to the nature of the NIS database.

Patients with AF and dementia have higher mortality and a lower likelihood of getting catheter ablation and electrical cardioversion. Females are also less likely to get catheter ablation and electrical cardioversion, even though they have similar in-hospital mortality compared to males. More research to understand sex-based disparities and differences based on dementia is needed to guide interventions to improve outcomes in females and patients with dementia.

## Funding

Baral, Nischit – None, Mitchell - None, Paul – None
Aggarwal - U24AG057437, P30AG010161, 1R01AG062637-01A1, 1R01AG054476-01A1, R01AG052583, R01AG056653, R01AG058679, R01 AG062689-02, 10.13039/100000957Alzheimer's Association - Interdisciplinary Summer Research Institute (Consultant)
Tracy - None, Seri – None, Arida - None, Sud - None, Kunadi - None
Baral, Nisha - None, Adhikari - None, Bashyal - None
Volgman - 10.13039/100000002NIH IND Number 119127; NIH NINR R01NR018443.

## CRediT authorship contribution statement

Nischit Baral: Conceptualization, methodology, software, writing-original draft preparation, editing.
Joshua D. Mitchell: Conceptualization, methodology, software, writing-reviewing and editing, supervision, validation, resources
Neelum T. Aggarwal: Conceptualization, writing-reviewing and editing
Timir K. Paul: Writing-reviewing and editing, validation, supervision
Amith Seri: Writing-reviewing and editing, software
Abdul K. Arida: Writing-reviewing and editing, software
Parul Sud: Writing-reviewing and editing, resources
Arvind Kunadi: Writing-reviewing and editing, resources
Krishna P. Bashyal: Writing-reviewing and editing,
Nisha Baral: Writing-reviewing and editing,
Govinda Adhikari: Writing-reviewing and editing
Melissa Tracy: Writing-reviewing and editing, Supervision, software, validation, resources
Annabelle Santos Volgman: Conceptualization, writing-reviewing and editing, Supervision, software, validation, resources

## Declaration of competing interest

Volgman – Sanofi (consulting), Pfizer (consulting), Merck (Consulting), Janssen (consulting), Bristol Myers Squibb Foundation Diverse Clinical Investigator Career Development Program (DCICDP), National Advisory Committee (NAC), Novartis Clinical Trial – Horizon Study, Apple Inc. stock.
Mitchell – Pfizer (consulting), BridgeBio (consulting). Research support from Pfizer, Bristol Meyers Squibb, Myocardial Solutions, Abbott Laboratories, and the Children's Discovery Institute are all unrelated to the manuscript's contents.

## Appendix A Supplementary data

Supplementary material: A. Fib and Dementia. Image 1
