# Supplementary material for: Sex-based disparities and in-hospital outcomes of patients hospitalized with atrial fibrillation with and without dementia
Source: Am Heart J Plus. 2023 Feb 3;26:100266. doi: 10.1016/j.ahjo.2023.100266 (PMC10945904; doi:10.1016/j.ahjo.2023.100266)
Supplement: Supplementary file 1 — Supplementary material: A. Fib and Dementia. [file mmc1.docx]

| Diagnosis/Procedure | ICD-10-diagnostic code OR ICD-10- procedural code used in the study |
| --- | --- |
| Atrial Fibrillation | I48.0, I48.1, I48.2, I48.3, I48.4, and I48.9, |
| Dementia | F01.5, F01.50, F01.51, F02.8, F02.80, F01.81, F03.9, F03.90, F03.91, F10.27, F10.97, F13.27, F13.97, F18.17, F18.27, F18.97, F19.17, F19.27, F19.97, G31.0, G31.09, G31.83 |
| Cardiogenic shock | R57.0 |
| Nontraumatic intracerebral and sub-arachnoid hemorrhage | I60.xx, I61.x |
| GI bleed excluding ulcerative colitis, Crohn’s disease, GI anomaly, infection, and GI cancers | K92.0, K92.1, K92.2 |
| Catheter ablation | 02583ZZ |
| Electrical cardioversion | 5A2204Z |
| Mechanical ventilation | 5A1955Z, 5A1945Z |
| Mechanical circulatory support (ECMO, Impella, Intra-aortic balloon pump) | 02HA0RS, 02HA3RS, 02HA4RS, 5A02116, 02HA0RZ, 02HA3RZ, 02HA4RZ, 02HA0RJ, 02HA3RJ, O2HA4RJ, 5A0211D, 5A0221D |
| Left Ventricular Assist Device | 02HA0QZ |

Abbreviation:

ICD-10: International Classification of Disease tenth revisions GI: Gastro-Intestinal

ECMO: Extra-Corporeal Membrane Oxygenation

eTable1: The ICD-10-CM diagnosis codes utilized for diagnosing Atrial fibrillation and Dementia from National Inpatient Sample 2016-2019


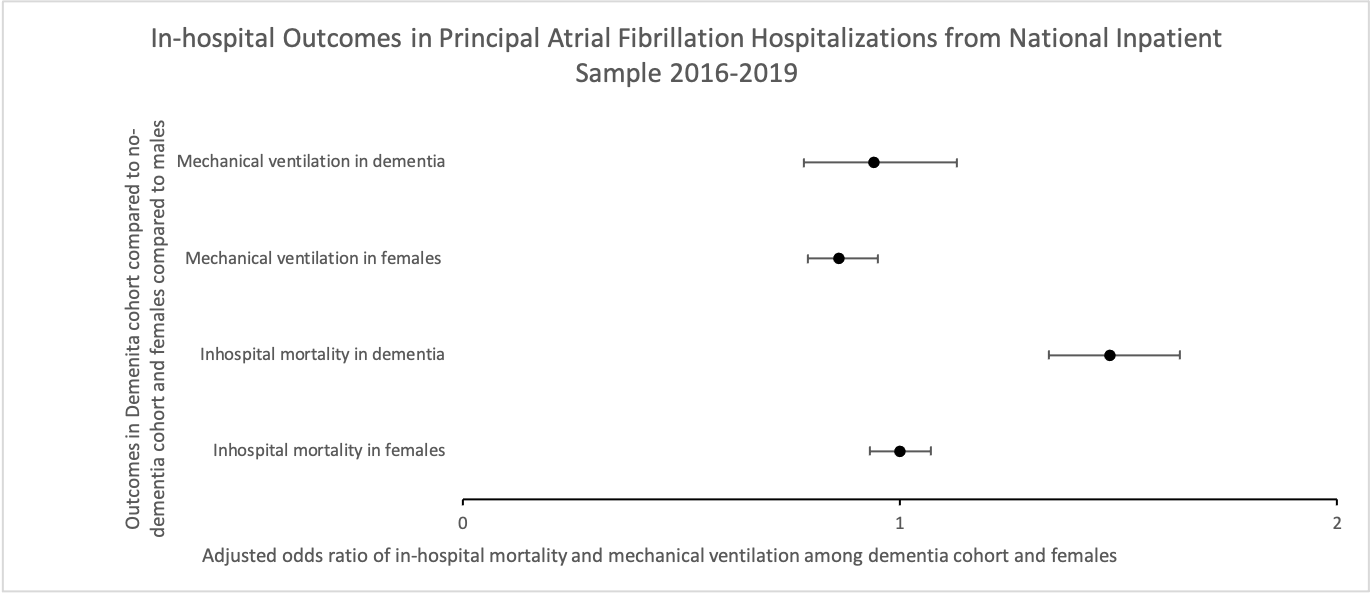


eFigure 1: Forest plot showing adjusted odds ratio of in-hospital mortality and mechanical ventilation procedure requirement among dementia cohort and females.


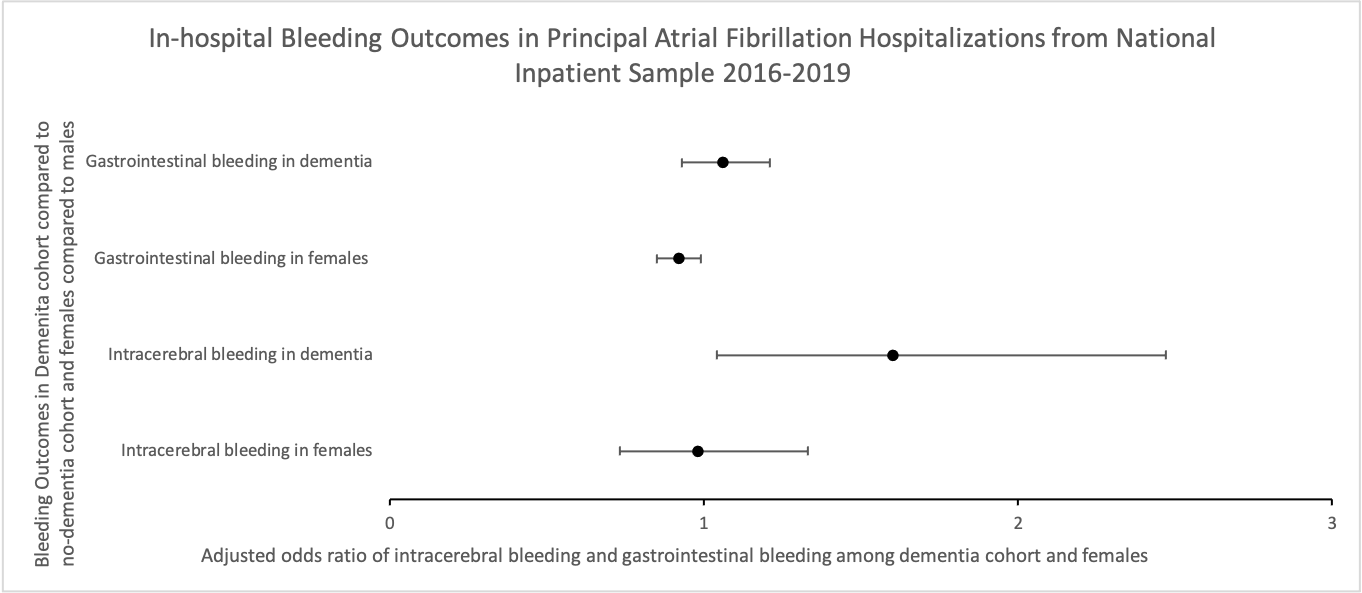


eFigure 2: Forest plot showing adjusted odds ratio of intracerebral bleeding and gastrointestinal bleeding among dementia cohort and females.


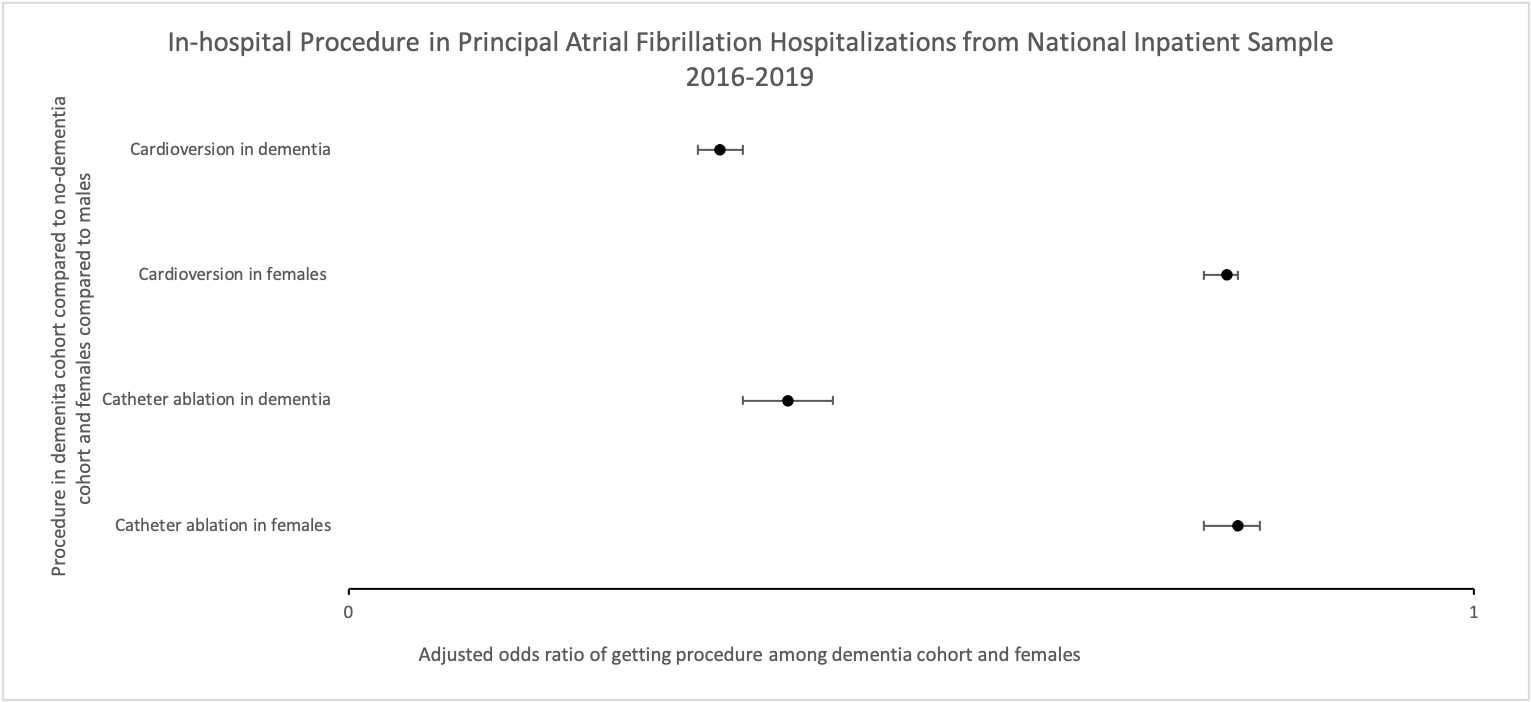


eFigure 3: Forest plot showing adjusted odds ratio of getting cardioversion and catheter ablation among dementia cohort and females.

**Abbreviations**

AF: Atrial Fibrillation

AIDS: Acquired Immunodeficiency Syndrome

AMI: Acute Myocardial Infarction

ARIC: Atherosclerosis Risk in Communities

ARIC-NCS: Atherosclerosis Risk in Communities-Neurocognitive Study

CEVD: Cerebrovascular Disease

CHF: Congestive Heart Failure

CI: Confidence Interval

COPD: Chronic Obstructive Pulmonary Disease

CVD: cardiovascular disease

DM: Diabetes Mellitus,

HMO: Health Maintenance Organization

LOS: Length of Stay

MCI: Mild Cognitive Impairment

NIS: National Inpatient Sample

PVD: Peripheral Vascular Disease

US: United States
